# A multicenter, randomized controlled trial comparing the identification rate of stigmata of recent hemorrhage and rebleeding rate between early and elective colonoscopy in outpatient-onset acute lower gastrointestinal bleeding: study protocol for a randomized controlled trial

**DOI:** 10.1186/s13063-018-2558-y

**Published:** 2018-04-03

**Authors:** Ryota Niikura, Naoyoshi Nagata, Atsuo Yamada, Hisashi Doyama, Yasutoshi Shiratori, Tsutomu Nishida, Shu Kiyotoki, Tomoyuki Yada, Tomoki Fujita, Tetsuya Sumiyoshi, Kenkei Hasatani, Tatsuya Mikami, Tetsuro Honda, Katsuhiro Mabe, Kazuo Hara, Katsumi Yamamoto, Mariko Takeda, Munenori Takata, Mototsugu Tanaka, Tomohiro Shinozaki, Mitsuhiro Fujishiro, Kazuhiko Koike

**Affiliations:** 1 Department of Gastroenterology, Graduate School of Medicine, The University of Tokyo, 7-3-1 Hongo, Bunkyo-ku, Tokyo, 1138655 Japan; 2 Department of Gastroenterology and Hepatology, National Center for Global Health and Medicine, Shinjuku-ku, Tokyo, Japan; 3 Department of Gastroenterology, Ishikawa Prefectural Central Hospital, Kanazawa-shi, Ishikawa Japan; 4 Department of Gastroenterology, St. Luke’s International Hospital, Chuo-ku, Tokyo, Japan; 5 Department of Gastroenterology, Toyonaka Municipal Hospital, Toyonaka-shi, Osaka, Japan; 6 Department of Gastroenterology, Shuto General Hospital, Yanai-shi, Yamaguchi Japan; 7 Department of Gastroenterology and Hepatology, National Center for Global Health and Medicine Kohnodai Hospital, Ichikawa-shi, Chiba Japan; 8 Department of Gastroenterology, Otaru Ekisaikai Hospital, Otaru-shi, Hokkaido Japan; 9 The Center for Digestive Disease, Tonan Hospital, Sapporo-shi, Hokkaido Japan; 10 Department of Gastroenterology, Fukui Prefectural Hospital, Fukui-shi, Fukui Japan; 11 Division of Endoscopy, Hirosaki University Hospital, Hirosaki-shi, Aomori Japan; 12 Department of Gastroenterology, Nagasaki Harbor Medical Center City Hospital, Nagasaki-shi, Nagasaki, Japan; 13 Department of Gastroenterology, National Hospital Organization Hakodate Hospital, Hakodate-shi, Hokkaido Japan; 14 Department of Gastroenterology, Aichi Cancer Center Hospital, Nagoya-shi, Aichi Japan; 15 Department of Gastroenterology, Japan Community Healthcare Organization Osaka Hospital, Osaka-shi, Osaka, Japan; 16 Clinical Research Support Center, The University of Tokyo Hospital, Bunkyo-ku, Tokyo, Japan; 17 Department of Biostatistics, School of Public Health, The University of Tokyo, Bunkyo-ku, Tokyo, Japan; 18 Endoscopy and Endoscopic Surgery, The University of Tokyo Hospital, Bunkyo-ku, Tokyo, Japan

**Keywords:** Early colonoscopy, Elective colonoscopy, Stigmata of recent hemorrhage, Acute lower gastrointestinal bleeding, Hemostasis, Rebleeding

## Abstract

**Background:**

The clinical benefit of early colonoscopy within 24 h of arrival in patients with severe acute lower gastrointestinal bleeding (ALGIB) remains controversial. This trial will compare early colonoscopy (performed within 24 h) versus elective colonoscopy (performed between 24 and 96 h) to examine the identification rate of stigmata of recent hemorrhage (SRH) in ALGIB patients. We hypothesize that, compared with elective colonoscopy, early colonoscopy increases the identification of SRH and subsequently improves clinical outcomes.

**Methods:**

This trial is an investigator-initiated, multicenter, randomized, open-label, parallel-group trial examining the superiority of early colonoscopy over elective colonoscopy (standard therapy) in ALGIB patients. The primary outcome measure is the identification of SRH. Secondary outcomes include 30-day rebleeding, success of endoscopic treatment, need for additional endoscopic examination, need for interventional radiology, need for surgery, need for transfusion during hospitalization, length of stay, 30-day thrombotic events, 30-day mortality, preparation-related adverse events, and colonoscopy-related adverse events. The sample size will enable detection of a 9% SRH rate in elective colonoscopy patients and a SRH rate of ≥ 26% in early colonoscopy patients with a risk of type I error of 5% and a power of 80%.

**Discussion:**

This trial will provide high-quality data on the benefits and risks of early colonoscopy in ALGIB patients.

**Trial registration:**

UMIN-CTR Identifier, UMIN000021129. Registered on 21 February 2016; ClinicalTrials.gov Identifier, NCT03098173. Registered on 24 March 2017.

**Electronic supplementary material:**

The online version of this article (10.1186/s13063-018-2558-y) contains supplementary material, which is available to authorized users.

## Background

Colonoscopy is essential for the diagnosis and treatment of patients hospitalized for acute lower gastrointestinal bleeding (ALGIB) [1]. The most important issue with diagnostic colonoscopy for ALGIB is infrequent identification of stigmata of recent hemorrhage (SRH) [2], which lowers the success of hemostasis. If SRH could be definitively identified enabling clinicians to perform accurate hemostasis, adverse clinical outcomes, such as rebleeding or transfusion requirement, could be reduced in ALGIB. An observational study found a positive association between early timing of colonoscopy and the identification of SRH [2]; however, it remains controversial as to whether an early procedure improves clinical outcomes [3, 4]. Although almost nearly half of all hospitals with a high colonoscopy procedure volume perform early colonoscopy, which is defined as being performed within 24 h, other hospitals perform later elective colonoscopy after full bowel preparation as standard care [5].

Recently, three meta-analyses [6–8] that included two single-center randomized controlled trials (RCT) [3, 4] for early versus elective colonoscopy in patients with ALGIB, showed that early colonoscopy has the potential to increase the identification of SRH and decrease the length of stay and associated costs; however, the meta-analyses also that early colonoscopy did not improve outcomes such as transfusion requirement, rebleeding, or mortality [6–8]. Nevertheless, these RCTs were stopped during enrollment because of difficulties in reaching the prespecified sample size. In addition, one of the RCTs was performed between 1993 and 1995, when colonoscopic procedures had not been standardized [3]. Thus, high-quality evidence for the early timing of colonoscopy in ALGIB is required.

To address these issues, we are conducting a multicenter RCT to compare the clinical outcomes between patients receiving early versus elective colonoscopy in ALGIB patients.

## Methods

### Trial design

**Fig. 1:**
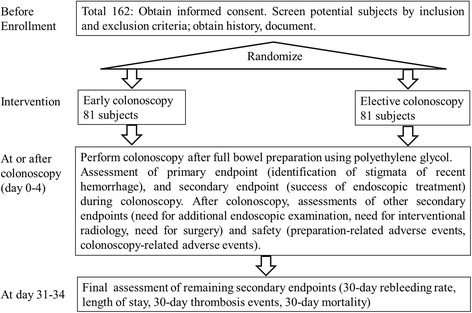
Schematic of study design

**Fig. 2:**
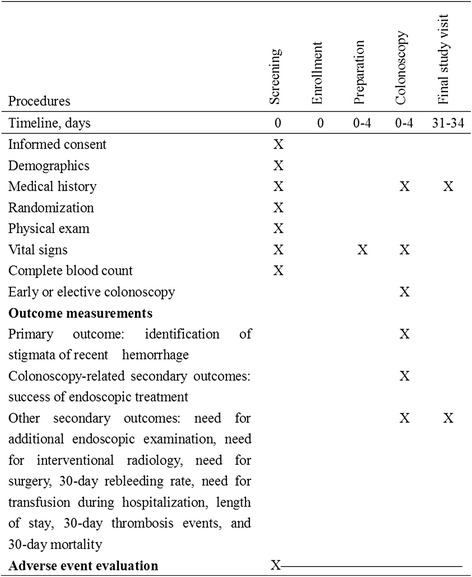
Timeline

This will be a parallel, randomized, open-label, superiority trial, in a 1:1 ratio, to receive early colonoscopy or elective colonoscopy (Figs. 1 and 2).

### Approvals

The trial has been approved by the institutional review board (IRB) of The University of Tokyo (P2015034–11Y, January 21, 2016) and the IRB of each participating institution. The trial is registered at UMIN-CTR (UMIN000021129, February 21, 2016) and ClinicalTrials.gov (NCT03098173, March 24, 2017).

### Setting

The study will include 15 Japanese hospitals with a high colonoscopy procedure volume.

### Population

#### Inclusion criteria

- Male or female outpatients aged ≥ 20 years who present with moderate-to-severe hematochezia or melena within 24 h of arrival, defined as (1) ≥ 3 occurrences of hematochezia within 8 h, or (2) hemorrhagic shock, or (3) requiring transfusion
- Eligible patients will be asked to read explanatory documents providing doctor’s instructions and sign consent forms. Informed consent will be obtained from each eligible patient before enrollment in the trial
- Stated willingness to comply with all study procedures and availability for the duration of the study

#### Exclusion criteria

- Hematemesis, black grounds vomitus
- Upper gastrointestinal bleeding, diagnosed by nasogastric tube or upper endoscopy
- Ingestion of the oral bowel preparation solution is not deemed possible
- Computed tomography performed before colonoscopy
- Diagnosis of peptic ulcer disease within the previous 10 days
- Ulcerative colitis or Crohn’s disease
- Abdominal surgery within the previous 10 days
- Polypectomy, endoscopic mucosal resection, or endoscopic submucosal dissection of the lower gastrointestinal tract within the previous 10 days
- Suspected perforation or peritonitis
- Suspected intestinal obstruction
- Hemorrhagic shock refractory to infusion or blood transfusion
- History of total colectomy
- Suspected disseminated intravascular coagulation
- End-stage malignant disease
- Severe cardiac failure
- Active thrombosis
- Severe respiratory failure
- Pregnancy

### Trial intervention

Enrolled patients will be randomized to undergo either early or elective colonoscopy. Early colonoscopy will be performed within 24 h of the initial visit. Elective colonoscopy will be performed between 24 and 96 h after the initial visit. All colonoscopies will be performed using an electronic video endoscope (Olympus Optical, Tokyo, Japan or Fujifilm Corporation, Tokyo, Japan) after administration of 2–4 L of oral bowel preparation. An enema will be additionally administered to patients who do not ingest all of the oral bowel preparation solution and continue to pass the effluent is free of fecal material.

### Outcome measures

#### Primary outcome measure

Identification of SRH in the lower gastrointestinal tract. We will define SRH based on colonoscopic visualization of lesions. These lesions include diverticulosis, tumor, ulcer, hemorrhoid, angioectasia, or polyp exhibiting active bleeding [9, 10], visible vessels [9, 11], and adherent clots [12]. We will also use endoscopic images to evaluate inter-observer agreement in SRH diagnoses between site investigators and an Independent-Effect Judgment Committee.

#### Secondary outcome measures

- Success of endoscopic treatment
- Need for additional endoscopic examinations
- Need for interventional radiology
- Need for surgery
- 30-day rebleeding rate
- Need for transfusion during hospitalization
- Length of stay
- 30-day thrombosis events
- 30-day mortality
- Preparation-related adverse events (AEs)
- Colonoscopy-related AEs (hemorrhagic shock and perforation)

### Outcome definitions

Outcome definitions are provided in greater detail in Additional file 1: Appendix S1.

### Screening

All patients referred to participating sites will be considered for screening and will be eligible if they fulfil all inclusion criteria and no exclusion criteria. Patients included and excluded will be reported according to the protocol [13].

### Randomization

Physicians at participating sites will have 24-h access to a web-based central randomization that provides immediate and concealed allocation. Randomization will be performed in blocks, with block sizes varying according to the allocation sequence generated by the Central Coordinating Unit, The University of Tokyo. A unique patient identification number will be entered into the system to ensure anonymity.

### Blinding

Not applicable.

### Safety

Participants will be free to withdraw from participation at any time upon request.

An investigator may terminate participation in the study if:

- A participant meets a newly developed or not previously recognized exclusion criterion that precludes further participation
- A clinical AE or other medical condition or situation occurs where continuing participation would not be in the participant’s best interests

The IRB of The University of Tokyo may terminate the trial in the event of a safety problem.

### Serious adverse event (SAE)

In compliance with the International Conference on Harmonisation (ICH) E6(R2) and Ethical Guidelines for Medical and Health Research Involving Human Subjects (Japan), investigators and monitoring personnel will consider an AE or suspected adverse reaction to be ‘serious’ if it results in any of the outcomes of death, a life-threatening AE, inpatient hospitalization or prolongation of hospitalization, persistent or significant incapacity, or substantial disruption of normal life functions*.*

All deaths and immediately life-threatening events, whether related or unrelated, will be recorded on the SAE form in the Electronic Data Capture (EDC) system and submitted to the IRB as soon as possible. All SAE information must be shared via e-mail or telephone with all investigators within 24 h of site awareness. If there is reasonable possibility that the study procedure caused an unanticipated SAE, the director of The University of Tokyo Hospital will report the SAE to the Ministry of Health, Labour and Welfare, Japan. All SAEs will be collected during study visits, interviews with study participants presenting for medical care, or reviews by study monitors and should be followed to adequate resolution.

### Statistics

#### Hypotheses and data analysis

- Primary efficacy endpoint is identification of SRH. The null hypothesis is that the SRH identification rates in patients undergoing early and elective colonoscopy are equal
- Secondary efficacy endpoints are success of endoscopic treatment, need for additional endoscopic examination, need for interventional radiology, need for surgery, need for transfusion during hospitalization, 30-day rebleeding rate, preparation-related AEs, colonoscopy-related AEs, 30-day thrombosis events, 30-day mortality, and length of stay; the null hypothesis is that the rates of the secondary endpoints in early and elective colonoscopy are equal

The study’s statistical and analytical plan has been decided in advance (Additional file 1: Appendix S2), and includes a more detailed analysis of the trial population and summary of statistical strategies to be used.

The primary analysis will include a ‘modified’ intention-to-treat population, excluding (1) patients who did not satisfy the enrollment criteria after randomization (i.e., patients who meet exclusion criteria, including withdrawal of consent, as assessed by the investigators), (2) patients who provided no post-randomization data on primary outcome (identification of SRH), and (3) patients who did not undergo colonoscopy, from a genuine intention-to-treat analysis set. The data on identification of SRH will be compared using the χ^2^ test in modified intention-to-treat population and the results will be presented as prevalence rate and number-needed-to-treat (number-needed-to-perform colonoscopy).

A secondary analysis will be conducted with the modified intention-to-treat population to compare the secondary outcomes of success of endoscopic treatment, need for additional endoscopic examination, need for interventional radiology, need for surgery, 30-day rebleeding rate, need for transfusion during hospitalization, length of stay, 30-day thrombosis events, 30-day mortality, preparation-related AEs, and colonoscopy-related AEs.

A sensitivity analysis will be conducted with the per-protocol population, subgroups of patients with colonic diverticular bleeding, patients terminated from the trial because of inadequate bowel preparation, patients who underwent endoscopic hemostasis, patients with colonic diverticular bleeding and who underwent endoscopic hemostasis, patients who underwent colonoscopy by an expert, patients who underwent colonoscopy within 24 h of onset of hematochezia, and each participating site.

Missing data will be removed in the primary analysis, and primary endpoint analysis will be performed by a complete case analysis. In the sensitivity analysis, primary endpoint analysis will be performed by imputation. Models and auxiliary variables for the imputation will be assessed by the trial investigators after locking the dataset.

For inferential tests, the *P* value for statistical significance will be < 0.05, two-tailed.

### Sample size estimation

Assuming that the SRH rate in the elective-colonoscopy patients is 9% and the SRH rate in the early-colonoscopy patients is 26% (or higher) [14], with an alpha level of 5% (two-sided), a sample size of 142 (2 × 71) patients will be required to ensure an 80% probability of obtaining a statistically-significant χ^2^ test result (i.e., an 80% statistical power). Because the observed difference might be diminished by patient noncompliance and/or dropout, we will recruit 20 additional patients to correct for these effects, and thus will recruit a total of 162 patients for this trial.

### Interim analyses

Interim analyses will not be done.

### Data registration

Data will be entered into a web-based EDC system by trial investigators or site investigators. The trial database will be created from the EDC system.

### Data handling and retention

Research data collected from participants for the purpose of statistical analysis and scientific reporting will be transmitted to and stored at the University of Tokyo Hospital. The study data entry system and the study management system to be used by the investigators at the clinical sites and the research staff at the University of Tokyo Hospital will be secure and password protected. At the end of the study, all study databases will be anonymized and archived at the University of Tokyo Hospital. Data will be retained for either a minimum of 5 years after the end of the study or for 3 years after publication, whichever is first.

### Monitoring

The trial monitoring plan is provided in Additional file 1: Appendix S3. The trial will be monitored by the risk-based monitoring team, consisting of the principal investigator, project manager, medical monitor, data managers, and a biostatistician, based at the University of Tokyo Hospital. Monitoring will consist of an early targeted review of certain data, which will include onsite, centralized, statistical monitoring for initial assessment and training.

### Ethical justification

The trial will adhere to ICH E6 (R2) and Ethical Guidelines for Medical and Health Research Involving Human Subjects (Japan) (Additional file 2). Recruitment can start following approval by the IRB of each participating site.

Early colonoscopy for ALGIB remains controversial and may be argued for by both patients and clinicians. The focus of this trial on early colonoscopy was decided based on the clinical uncertainty that exists in daily practice.

- No high-quality evidence supports the suggestion that early colonoscopy improves the identification of SRH or clinical outcomes over those achieved with elective colonoscopy in ALGIB patients; this controversial clinical question should therefore be addressed
- If this study finds that early colonoscopy is beneficial for ALGIB patients, fewer transfusions, lower rebleeding rates, and shorter hospital stays may result; in such case, early colonoscopy will become more widespread in clinical practice
- An observational study reported that early colonoscopy was performed in 40% of ALGIB patients [10]; another questionnaire survey in 37 major hospitals in Japan showed that 64% of these hospitals performed early colonoscopy [5]; in clinical practice, early colonoscopy in ALGIB is feasible for many endoscopists
- A Japanese observational study showed that colonoscopy in ALGIB patients did not increase AEs compared with colonoscopy in non-gastrointestinal bleeding patients [15]

### Enrolment

Patients from Tokyo, Ishikawa, Chiba, Hokkaido, Osaka, Fukui, Aomori, Aichi, Nagasaki, and Yamaguchi prefectures in Japan are expected to participate in the trial. The trial was initiated in Tokyo, Japan, in July 2016 and participation by the other sites can follow now that IRB approval has been obtained. Recruitment is expected to take 3 years.

### Trial management and organization

The trial is supported by the Clinical Research Support Center at the University of Tokyo Hospital and the Department of Biostatistics, School of Public Health at the University of Tokyo.

An Independent Effect Judgment Committee has been formed (Additional file 1: Appendix S4). For the primary endpoint, its role is to evaluate inter-observer agreement in SRH diagnoses between site investigators and its Committee members.

A Site Investigator Team, consisting of a principal investigator and a trial coordinator at each site, will manage and coordinate the trial locally. The principal investigator at each site is responsible for data collection and maintenance of trial documentation.

### Publication

Upon completion of the trial, a main manuscript will be prepared to present the trial results –positive, negative, or neutral – and will be submitted for peer-review to a major clinical journal. The results will also be published at ClinicalTrials.gov and on the website of the Department of Gastroenterology, the University of Tokyo.

### Data sharing

A file containing the clean dataset used for final analysis to determine the main results of the trial, the statistical analysis plan, an explanation of variables, and the study protocol will be made publicly accessible in an anonymized format.

### Timeline

2015–2016: Funding applications, ethical approvals, EDC system development, monitoring plan development, and clinical staff training.

2016–2019: Patient recruitment.

2019: Data analysis and writing and submission of the main manuscript for publication.

### Collaborators

This trial was developed and will be conducted in collaboration with all participating sites. The web-based randomization system and the system for allocation were developed and will be administered by the Clinical Research Support Center, the University of Tokyo Hospital.

## Discussion

### Rationale

Two premature RCTs [3, 4] and one propensity matching analysis [14], which was performed at a single center, have been published on investigations of whether early colonoscopy improved identification rates of SRH and clinical outcomes. One of the RCTs, an open-label trial by an American study group [3], compared early and elective colonoscopy in 100 ALGIB patients and reported that early colonoscopy had a higher identification of SRH than that with elective colonoscopy, although it did not improve clinical outcomes, including rebleeding, transfusion, and mortality. In contrast, the second RCT, an open-label trial by another American study group [4], compared early and elective colonoscopy in 72 ALGIB patients and reported no difference in identification rate of SRH, rebleeding, transfusion, or length of stay. However, both trials were terminated before reaching the originally planned sample size and were unable to demonstrate the superiority of early colonoscopy. In the study involving propensity matching analysis, we performed the largest retrospective propensity-score-matched analysis to compare identification rates of SRH and clinical outcomes (including success of endoscopic hemostasis, 30-day rebleeding, and length of stay) between early and elective colonoscopy [14]. Early colonoscopy was associated with a higher identification rate of SRH (26%) compared with elective colonoscopy (9%), as well as with a higher success rate for endoscopic hemostasis and shorter length of stay; however, the significance of the findings were limited by unmeasured confounders. These findings further highlighted the need for a multicenter RCT to determine the benefits and risks of early colonoscopy in ALGIB.

### Outcome

In this trial, we focus on the identification of SRH as the most important and feasible primary outcome. Although SRH identification may be a surrogate for clinical outcomes such as rebleeding or death, we hypothesized that early colonoscopy increases the identification of SRH and enables adequate endoscopic treatment, leading to fewer cases of rebleeding, less transfusion requirement, and shorter hospital stays [6–8]. Another reason is sample size – if the sample size calculation were based on a 6% difference in the rate of 30-day rebleeding between early and elective colonoscopy (13% vs. 7%) [14], quite a large sample size of 784 patients (392 per group) would be needed, and conducting RCTs in this situation may be challenging considering that a prior RCT took 6 years to complete in an emergency setting of ALGIB [4].

### Strengths

This trial will build upon collaborations between major hospitals in Japan to deliver a study that may begin to inform the rational use of early colonoscopy for patients admitted with ALGIB. The RCT study design is justified in order to demonstrate that early colonoscopy can be implemented at a hospital-wide level, to reduce contamination between trial interventions, and to aid in the operational aspects of trial delivery. This is ethically acceptable, given that both early and elective colonoscopies are within the realms of normal practice in Japan and that all clinicians have the discretion to perform a colonoscopy in contravention of the policy if they think it is necessary, thereby ensuring patient safety is not compromised. We believe the study may also help inform the wider debate about the use of early colonoscopy.

### Limitations

Open label study.

### Perspective

Together with existing data, the high-quality data provided by this trial will inform clinicians and guideline committee members on the utility of early colonoscopy in ALGIB patients, as well as accumulate evidence for its use in moderate to severe outpatient cases of ALGIB.

### Trial status

Recruitment is planned to end by March 2019.

## Additional files

Additional file 1: **Appendix S1.** Outcome Definitions. **Appendix S2.** Statistical analysis plan. **Appendix S3.** Clinical data monitoring plan. **Appendix S4.** Key role. (DOCX 57 kb)

Additional file 2: SPIRIT 2013 Checklist: Recommended items to address in a clinical trial protocol and related documents*. (DOC 121 kb)

## Abbreviations

AE: adverse event
ALGIB: acute lower gastrointestinal bleeding
EDC: electronic data capture
ICH: International Conference on Harmonisation
IRB: investigational review board
SAE: serious adverse event
SRH: stigmata of recent hemorrhage
