# Supplementary material for: A multicenter, randomized controlled trial comparing the identification rate of stigmata of recent hemorrhage and rebleeding rate between early and elective colonoscopy in outpatient-onset acute lower gastrointestinal bleeding: study protocol for a randomized controlled trial
Source: Trials. 2018 Apr 3;19:214. doi: 10.1186/s13063-018-2558-y (PMC5883587; doi:10.1186/s13063-018-2558-y)
Supplement: Supplementary file 1 — Appendix S1. Outcome Definitions. Appendix S2. Statistical analysis plan. Appendix S3. Clinical data monitoring plan. Appendix S4. Key role. (DOCX 57 kb) [file 13063_2018_2558_MOESM1_ESM.docx]

# **Appendix S1. Outcome Definitions**

| Outcome | Definitions |
| --- | --- |
| Success of endoscopic treatment | Success will be defined as the number achieving hemostasis per total number of attempts at endoscopic hemostasis during colonoscopy examination. |
| Need for transfusion during hospitalization | Transfusion will be performed when the hemoglobin level falls to < 7 g/dL in patients, according to the guidelines of the Ministry of Health, Labour, and Welfare. |
| 30-day rebleeding rate | Rebleeding will be defined as significant fresh blood loss after an initial colonoscopy with any of the following criteria:  i) Hemorrhagic shock, including cold sweat, nausea, syncope, or systolic blood pressure ≤ 90 mmHg.  ii) Need for transfusion, according to the guidelines of the Ministry of Health, Labour, and Welfare.  iii) Further colonoscopy identifies blood pooling, or  iv) SRH in the lower gastrointestinal tract.  v) Contrast-enhanced CT identifies extravasation in the colorectal region.  However, these examinations will not be performed routinely if rebleeding occurs in the study period. |
| 30-day thrombosis events | Thrombosis events will include acute coronary syndromes, including angina pectoris and myocardial infarction, stroke, including cerebrovascular infarction, cerebral hemorrhage, and transient ischemic attacks, deep vein thrombosis, and pulmonary embolism. |
| Preparation-related adverse events | Preparation-related adverse events will include nausea, vomiting, abdominal pain, volume overload, aspiration pneumonia, hemorrhagic shock, exacerbation bleeding, and ileus |
| Colonoscopy-related adverse events | Colonoscopy-related adverse events will include hemorrhagic shock, and perforation. |

# **Appendix S2. Statistical Analysis Plan**

**Statistical Analysis Plan**

**A multi-center, randomized controlled trial comparing early versus elective colonoscopy in outpatients with acute lower gastrointestinal bleeding**

**Version**: 2.0

**Author**: Tomohiro Shinozaki, Department of Biostatistics, School of Public Health, the University of Tokyo

**Date**: 9-1-2018

**1. INTRODUCTION**

Acute lower gastrointestinal bleeding (ALGIB) is a common disease, the incidence of which has recently increased.^1^ ‘Early’ colonoscopy, performed within 24 h of arrival, potentially improves clinical outcomes, such as the identification of stigmata of recent hemorrhage (SRH) and rebleeding compared with ‘elective’ colonoscopy, performed between 24 and 96 h.

However, we have identified a lack of high-quality evidence regarding the optimal timing of colonoscopy in ALGIB.^2-4^ There is widely varying clinical use of early colonoscopy throughout Japan, and a randomized controlled trial (RCT) in a patient population will address a key area of clinical uncertainty.^6^

This trial may begin to inform the rational use of early colonoscopy for patients admitted with ALGIB. A RCT design is justified to demonstrate that early colonoscopy can be implemented at a hospital-wide level, to reduce contamination between the trial interventions, and to aid in operational aspects of the trial delivery. This is acceptable ethically, given that both ‘early’ and ‘elective’ colonoscopies are within the realms of normal practice in Japan and that all clinicians have the discretion to perform a colonoscopy in contravention of the policy if they think it is necessary, thereby ensuring patient safety is not compromised.^5^ We believe the study may also help to inform the wider debate regarding the use of early colonoscopy.

The objective of the study is to compare the SRH identification rates of ‘early’ versus ‘elective’ colonoscopy in outpatients with ALGIB.

**2 DATA SOURCE**

All data to be analyzed are obtained from “A multi-center, randomized controlled trial comparing early versus elective colonoscopy in outpatients with acute lower gastrointestinal bleeding.” Variables to be measured are specified in the protocol. Datasets are produced in compliance with the Clinical Data Monitoring Plan.

**3 ANALYSIS OBJECTIVES**

- Primary efficacy endpoint: The rate of identification of stigmata of recent hemorrhage (SRH).

Null hypothesis: The SRH identification rates in patients undergoing early and elective colonoscopy are equal.

- Secondary efficacy endpoints: Success of endoscopic treatment, need for additional endoscopic examination, need for interventional radiology, need for surgery, need for transfusion during hospitalization, 30-day rebleeding rates, preparation related adverse events, colonoscopy-related adverse events, 30-day thrombosis events, 30-day mortality, and length of stay.

Null hypothesis: The rates of the secondary endpoints in early and elective colonoscopy are equal.

**4 ANALYSIS SETS/ POPULATIONS/SUBGROUPS**

- ANALYSIS SETS

The following two analysis sets are analyzed: modified intention-to-treat (ITT) and per-protocol analysis sets. A genuine ITT analysis set includes the data of all patients participating in the trial, but a modified ITT analysis set excludes patients 1) who did not fulfill the enrollment criteria after randomization (i.e. patients with exclusion criteria including withdraw consent) these criteria are assessed by investigators, 2) who provide no post-randomization data of primary outcome (identification of SRH), and 3) who do not undergo colonoscopy (i.e., ‘early’ nor ‘elective’) from a genuine ITT analysis set. The per-protocol analysis set includes the data on patients who 1) fulfill the enrollment criteria after randomization and 2) undergo early or elective colonoscopy.

- PARTICIPANT INCLUSION CRITERIA

1. Males or females outpatients aged ≥ 20 years, presenting with moderate-to-severe hematochezia or melena within 24 h of arrival, defined as (i) more than three occurrences of hematochezia within 8 h, or (ii) hemorrhagic shock, or (iii) requiring transfusion.
2. Eligible patients will be asked to read explanatory documents providing doctor’s instructions and sign consent forms. Informed consent will be obtained from each eligible patient before enrollment in the trial..
3. Stated willingness to comply with all study procedures and availability for the duration of the study.

- PARTICIPANT EXCLUSION CRITERIA

An individual who meets any of the following criteria will be excluded from participation in this study:

1. Patients with hematemesis, black vomiting.
2. Patients with upper gastrointestinal bleeding, diagnosed by nasogastric tube or upper endoscopy.
3. Patients who have impossible consumed the oral bowel preparation solution.
4. Patients who have undergone computed tomography.
5. Patients in whom peptic ulcer diseases have been diagnosed within the previous 10 days.
6. Ulcerative colitis or Crohn’s disease patients.
7. Patients who have undergone abdominal surgery within the previous 10 days.
8. Patients who have undergone polypectomy, endoscopic mucosal resection, or endoscopic submucosal dissection of the colon within the previous 10 days.
9. Patients with suspected perforation or peritonitis.
10. Patients with suspected intestinal obstruction.
11. Patients with hemorrhagic shock refractory to infusion or blood transfusion.
12. Patients who have undergone a total colectomy.
13. Patients with suspected disseminated intravascular coagulation.
14. Patients with end-stage malignant disease.
15. Patients with severe cardiac failure.
16. Patients with active thrombosis.
17. Patients with severe respiratory failure.
18. Pregnant patients.

- SUBGROUP ANALYSES

The primary endpoint is analyzed based on subgroups of patients with colonic diverticular bleeding, patients terminated for inadequate bowel preparation, patients who underwent endoscopic hemostasis, patients with colonic diverticular bleeding and who underwent endoscopic hemostasis, patients who underwent colonoscopy by an expert, each site, and patients who underwent colonoscopy within 24 h of onset of hematochezia. These subgroups include secondary interest which subgroup is most appropriate for early colonoscopy. ^6-8^ Nevertheless primary analyses fail to produce statistically significant results, subgroup analyses are performed.

**5 ENDPOINTS AND COVARIATES**

- PRIMARY ENDPOINT

Identification of SRH in lower gastrointestinal tract.

We define SRH based on colonoscopic visualization of lesions, such as diverticulosis, tumor, ulcer, hemorrhoid, angioectasia, and polyps exhibiting active bleeding, a visible vessel, or an adherent clot. We also evaluate inter-observer agreement of SRH diagnoses among site investigators and an independent-effect judgment committee using endoscopic images.

- SECONDARY ENDPOINTS

1. Success of endoscopic treatment
2. Need for additional endoscopic examination
3. Need for interventional radiology
4. Need for surgery
5. 30-day rebleeding rates
6. Need for transfusion during hospitalization
7. Length of stay
8. 30-day thrombosis events
9. 30-day mortality
10. Preparation-related adverse events
11. Colonoscopy-related adverse events

**6 HANDLING OF MISSING VALUES AND OTHER DATA CONVENTIONS**

The primary analysis for both primary and secondary endpoints, is performed by complete case analysis, which excludes patients whose data are missing. As a sensitivity analysis, missing data is substituted by a multiple imputation method. Models and auxiliary variables for the imputation are assessed by the trial investigators after fixing a dataset.

**7.1 STATISTICAL PROCEDURES**

- ANALYSIS OF THE PRIMARY EFFICACY ENDPOINT

Definition of the measurement: Identification of SRH in lower gastrointestinal tract.

The scale: Binary/categorical.

The χ^2^ test is used to analyze the primary endpoint, and results are presented prevalence rates and number needed to treat (number needed to perform colonoscopy).

- ANALYSIS OF THE SECONDARY ENDPOINTS

Definition of the measurement: success of endoscopic treatment, need for additional endoscopic examination, need for interventional radiology, need for surgery, 30-day rebleeding rates, need for transfusion during hospitalization, length of stay, 30-day thrombosis events, 30-day mortality, preparation-related adverse events, and colonoscopy-related adverse events. Each outcome is defined in the Appendix.

The scale:

Binary/categorical: success of endoscopic treatment, need for additional endoscopic examination, need for interventional radiology, need for surgery, 30-day rebleeding rates, need for transfusion during hospitalization, 30-day thrombosis events, 30-day mortality, preparation-related adverse events, and colonoscopy-related adverse events (hemorrhagic shock, and perforation).

Interval: Length of stay.

The χ^2^ test or Fisher’s exact test is used to analyze the secondary endpoints of success of endoscopic treatment, need for additional endoscopic examination, need for interventional radiology, need for surgery, 30-day rebleeding rates, need for transfusion during hospitalization, 30-day thrombosis events, 30-day mortality, preparation-related adverse events, and colonoscopy-related adverse events, as appropriate, and results are presented as prevalence rates and number needed to treat (number needed to perform colonoscopy).

Wilcoxon’s rank-sum test is used to analyze the secondary endpoint of length of stay. Results are presented as means with standard errors or medians with percentiles, or both.

- SAFETY ANALYSES

Safety endpoints (preparation- and colonoscopy-related adverse events) are analyzed as summary statistics during preparation and colonoscopy.

Adverse events (AEs) are coded based on the Medical Dictionary for Regulatory Activities/Japanese version (MedDRA/J)) and counted once only for a given participant. Evaluated start date, stop date, severity, relationship, outcome, and duration; and presented severity, frequency, and relationship of AEs to preparation and colonoscopy are presented by system organ class (SOC) and preferred term groupings.

- ADHERENCE AND RETENTION ANALYSES

Adherence measures of the performance of the allocated form of colonoscopy are assessed and calculated. Similarly, study retention/loss to follow-up, and frequency of, and reasons for, discontinuation of the intervention are assessed and calculated.

- BASELINE DESCRIPTIVE STATISTICS

For descriptive statistics, data is summarized by treatment group. Number, mean, standard deviation, minimum and maximum are summarized by continuous efficacy variables, whereas number and percent are summarized by categorical efficacy variables. Inferential statistics are not used.

**7.2 MEASURES TO ADJUST FOR MULTIPLICITY, CONFOUNDERS, HETEROGENEITY, ETC.**

Two confounders (patients who underwent colonoscopy by an expert and each participating site) are adjusted using a Mantel-Haenzsel test in the primary analysis. Mantel-Haenzsel risk differences and ratios are calculated for strata of i) patients who underwent colonoscopy by an expert, and ii) each participating site. Heterogeneity for each endpoint is assessed by subgroup analyses (as described in Section 4), using (approximate) interaction tests based on the difference in effect-measures among subgroups.

**8 SENSITIVITY ANALYSES**

As described in Section 6, sensitivity analysis for missing data is performed by a multiple imputation method.

**9 QC PLANS**

Quality control (QC) procedures will be implemented, beginning with the data entry system, and data QC checks that will be run on an electronic data capture (EDC) system will be generated. Any missing data or data anomalies will be communicated to the sites for clarification/resolution.

Following written standard operating procedures (SOPs), the monitors will verify that the clinical trial is conducted and data are generated, recorded, and reported in compliance with the protocol, *GCP*, and *Ethical Guidelines for Medical and Health Research Involving Human Subjects (Japan)*.

The investigational site will provide direct access to all trial-related sites, source documents, and reports for the purpose of monitoring and auditing by the principal investigator (PI), and inspection by local and regulatory authorities.

**10 PROGRAMMING PLANS**

A Statistician (TS) writes SAS code for all planned analyses before linking a dataset to randomization labels (i.e., ‘early’ versus ‘elective’). Statistical computations and figures in tables are generated using SAS software, version 9.4 (SAS, Cary, NC).

**11 REFERENCES**

1. Longstreth GF. Epidemiology and outcome of patients hospitalized with acute lower gastrointestinal hemorrhage: a population-based study. Am J Gastroenterol 1997;92:419-24.

2. Niikura R, Nagata N, Aoki T, et al. Predictors for identification of stigmata of recent hemorrhage on colonic diverticula in lower gastrointestinal bleeding. J Clin Gastroenterol 2015;49:e24-30.

3. Green BT, Rockey DC, Portwood G, et al. Urgent colonoscopy for evaluation and management of acute lower gastrointestinal hemorrhage: a randomized controlled trial. Am J Gastroenterol 2005;100:2395-402.

4. Laine L, Shah A. Randomized trial of urgent vs. elective colonoscopy in patients hospitalized with lower GI bleeding. Am J Gastroenterol 2010;105:2636-41.

5. Niikura R, Nagata N, Shimbo T, et al. Adverse Events during Bowel Preparation and Colonoscopy in Patients with Acute Lower Gastrointestinal Bleeding Compared with Elective Non-Gastrointestinal Bleeding. PLoS One 2015;10:e0138000.

6. Niikura R, Nagata N, Doyama H, et al. Current state of practice for colonic diverticular bleeding in 37 hospitals in Japan: A multicenter questionnaire study. World J Gastrointest Endosc 2016;8:785-794.

7. Strate LL, Naumann CR. The role of colonoscopy and radiological procedures in the management of acute lower intestinal bleeding. Clin. Gastroenterol. Hepatol 2010; 8: 333-343.

8. Strate LL, Orav EJ, Syngal S. Early predictors of severity in acute lower intestinal tract bleeding. Arch. Intern. Med. 2003; 163: 838-843.

# **Appendix S3.** **CLINICAL DATA MONITORING PLAN**

**CLINICAL DATA MONITORING PLAN**

**A multi-center, randomized controlled trial comparing early versus elective colonoscopy in outpatients with acute lower gastrointestinal bleeding**

Sponsor: Graduate School of Medicine, The University of Tokyo

Funded by: The Japanese Gastroenterological Association

Draft or Version Number: v.1.0. 9 June 2016

**1.****PURPOSE**

The purpose of this document is to specify all study-specific monitoring requirements for a multi-center, randomized controlled trial comparing early versus elective colonoscopy in outpatients with acute lower gastrointestinal bleeding protocol that ensures that the clinical sites comply with the study protocol and regulatory requirements.

**2．****TOOLS AND PROCESSES**

**2.1 Study Data**

This study will use direct data entry of clinical trial data. This process will allow a clinical study site to perform direct data entry of original data into an electronic data capture (EDC) system at the time of the subject’s hospital visit, and for the original data to be stored in the access-controlled data repository, access to which will be controlled by the clinical investigator. These original data will be stored in the Hospital Information System prior to the data being transmitted to the EDC database.

**3.** **Risk Mitigation Strategy**

| **Category** | **Risk** | **Impact** | **Probability** | **Detectability** | **RPE*** | **Risk Mitigation** |
| --- | --- | --- | --- | --- | --- | --- |
| Trial outcome | Missing identification rate of stigmata of recent hemorrhage | High | Medium | High | High | 100% SDV and SDR (on site monitoring) |
| Subject safety | Risk is greater in the elective group than early group | High | Low | High | Low | Planned sample size will provide sufficient statistical power including patients with protocol violations |
| Subjects safety | Specific reporting requirements for severe adverse events (SAE) | High | Low | High | High | When an SAE occurs, information is automatically transmitted from the EDC system to stakeholders |
| Subject registration | Violation of eligibility | High | Low | High | High | 100% SDV and SDR (onsite monitoring) |
| Subject registration | Allocation | High | Low | High | High | All allocation is performed by the EDC system |
| Subjects’ demographic data | Concomitant Medication | Low | Low | High | Low | Central monitoring confirms data inconsistency |
| Subjects’ demographic data | Past history of Illness | Low | Low | High | Low | Using Charson Comorbidity Index, central monitoring confirms data inconsistency |
| Procedure | Data collection | Low | Low | High | Low | Vital signs, laboratory data and other continuous variables are extracted from the EDC system; a biostatistician performs central statistical monitoring and detects inaccurate data |
| Procedure | Colonoscopy | Low | Low | High | Low | All facilities are endoscopic special facilities, which have many endoscopists to complete colonoscopy procedures in compliance with the protocol |
| Discontinuation | Discontinuation of study subjects | High | Low | High | High | Central monitoring confirms data |
| Facility selection | Facility selection for this study | Low | Low | Low | Low | All facilities have own institutional review board and share study aims. Thus, these facilities are able to recruit the planned number of subjects |

*****RPE, Risk priority number evaluation

**4. SOURCE DOCUMENTS**

1. Source data/records contain all information necessary for the reconstruction and evaluation of the study. Source data/records include original records, certified copies of original records, observations, and laboratory reports and/or data sheets. In addition, with the use of direct data entry, the access-controlled data repository will serve as an original record.

2. At the time of the first monitoring visit or during the initiation visit, the source of original data, whether it is being collected in electronic or paper format, will be identified for each site.

**5. MONITORING**

Onsite monitoring visits will focus on ensuring that the clinical site understands and is following the protocol, reviewing completeness and accuracy of informed consent forms, risk-based source document verification (SDV) of original records, and other issues that may occur during the course of the clinical trial.

Central monitoring will focus on assessment of the “reasonableness” of data entered into EDC system and data quality management metrics. Central statistical monitoring will focus on assessment of the veracity of data entered into the EDC system using statistical methods.

**5.1 Onsite Monitoring**

For each site, the responsible monitor will schedule the first onsite monitoring visit to confirm the informed consent (Day 0) of any of the first three subjects. Based on the findings at this visit, coupled with central monitoring findings, the monitor will decide when to schedule the next monitoring visit.

For each site, the monitor will schedule a monitoring visit immediately prior to, or coinciding with, the first subject’s final study visit (Day 31+/3). The purpose of this visit will be primarily to retrain the site personnel on the relevant study procedures.

Interim monitoring visits will include review of the following:

1. Informed consent process and forms (100%)
2. Study conduct and protocol adherence
3. Subject eligibility (100%)
4. Adverse events (100%)
5. Personnel delegation and signature log
6. Patient medical records
7. Protocol deviations and violations
8. Follow-up of outstanding issues
9. The certification process of data originally collected on paper and subsequently entered into the EDC system

Where a site maintains patient records that duplicate information captured in the EDC system, the monitor will review those records specified below, to ensure that the site records match those captured in the EDC system:

1. Demographics (100%). To ensure subject identities based on the site’s medical records.

2. Medical history. To ensure that sites have entered all relevant inclusion/exclusion criteria into the EDC system (100%).

3. Confirmation of subject’s visit to the clinical site (100% of first three subjects).

4. Review of office medical records (100% of first three subjects).

When findings indicate that retraining is required, the monitor will retrain site staff as soon as possible.

**5.2 Central Monitoring**

Data manager (DM) will perform central monitoring through data review and cleaning:

1. A 100% review of all entered forms and issue queries and, if needed.

2. Review and take appropriate action for all online and batch edit checks.

DM will review periodically the EDC for accuracy and completeness. Risk-based monitoring meetings will take place when 20, 40, 80, and 120 cases have accumulated, and will involve DM, monitor, biostatistician (as needed), to review the progress of the clinical trial. Items to be reviewed at the risk-based monitoring meetings may include:

1. Enrollment and dropout status
2. An assessment of edit checks and queries that are being filed, by form as well as by variable
3. Reasons for changes to the database by the clinical site
4. Adverse events
5. Medications
6. Protocol deviations and violations
7. Monitoring procedures
8. Other items that may arise
9. Action items

The project manager will record meeting minutes and follow-up actions. The schedule of meetings and the clinical monitoring plan may be modified depending on findings. The decisions and the rationale for changing any of the procedures will be documented.

**6. Startup meeting**

The purpose of a startup meeting is to train investigators and site personnel on the specific requirements and procedures needed to perform the clinical trial. The startup meeting will be held during the risk-based monitoring meeting, or if it is determined that a specific site requires additional training, as appropriate. Sites will not enroll subjects into the trial until the startup meeting has been satisfactorily completed.

At a minimum, the agenda for the startup meeting must include the following elements:

1. Review of the protocol

2. Training appropriate staff on:

1. GCP regulations
2. SAE reporting requirements
3. Subject management
4. Handling of colonoscopy examination
5. Handling of safety colonoscopy examination
6. EDC system
7. Certification of original records
8. Direct data entry process

**7. Interim Monitoring Visits**

The purpose of an interim monitoring visit will be to ensure that the rights and well-being of each subject are protected; trial data are accurate, complete, and verifiable; the trial is being conducted according to ICH GCP guidelines and Ethical Guidelines for Medical and Health Research Involving Human Subjects; and the trial site and staff remain trained and qualified. Monitoring of the clinical trial can occur both by onsite visits and through central monitoring procedures.

**8. Closeout Visit**

The purpose of a closeout visit will be to bring official completion to all trial-related activities at the site.

# **Appendix S4. Key role**

**Funder**

The Japanese Gastroenterological Association

Dr. Kazuma Fujimoto

The Japanese Gastroenterological Association Director

2-1-1 Suido, Bunkyo-ku, Tokyo

Telephone: +81-3-5840-6338 Email: jga-secretariat@keiso-comm.com

**Principal Investigator**

Dr. Kazuhiko Koike

Department of Gastroenterology, Graduate School of Medicine, The University of Tokyo

7-3-1 Hongo, Bunkyo-ku, Tokyo, Japan

Telephone: +81-3-3815-5411 Email: kkoike-tky@umin.ac.jp

Dr. Atsuo Yamada

Department of Gastroenterology, Graduate School of Medicine, The University of Tokyo

7-3-1 Hongo, Bunkyo-ku, Tokyo, Japan

Telephone: +81-3-3815-5411 Email: yamada-a@umin.ac.jp

**Project Manager**

Dr. Ryota Niikura

Department of Gastroenterology, Graduate School of Medicine, The University of Tokyo

7-3-1 Hongo, Bunkyo-ku, Tokyo, Japan

Telephone: +81-3-3815-5411 Email: rniikura@triton.ocn.ne.jp

**Site Investigator**

Dr. Naoyoshi Nagata

Department of Gastroenterology and Hepatology, National Center for Global Health and Medicine center hospital

1-21-1 Toyama, Shinjuku-ku, Tokyo, Japan

Telephone: +81-3-3202-7181 Email: nnagata_ncgm@yahoo.co.jp

Dr. Hisashi Doyama

Department of Gastroenterology, Ishikawa prefectural central hospital

Kuratsuki-Higashi 2-1, Kanazawa city, Ishikawa, Japan

Telephone: +81-76-237-8211 Email: doyama.134@ipch.jp

Dr. Yasutoshi Shiratori

Department of Gastroenterology, St. Luke’s International Hospital

9–1 Akashi-cho, Chuo-ku, Tokyo, Japan

Telephone: +81-3-3541-5151 Email: naoishi@luke.ac.jp

Dr. Tomoyuki Yada

Department of Gastroenterology and Hepatology, National Center for Global Health and Medicine kohnodai hospital

1-7-1 Kohnodai, ichikawa-shi, Chiba, Japan

Telephone: +81-47-372-3501 Email: tomoyuki0618@yahoo.co.jp

Dr. Tomoki Fujita

Department of Gastroenterology, Otaru Ekisaikai Hospital

1-4-1 Inaho, Otaru-shi, Hokkaido, Japan

Telephone: +81-134-24-0325 Email: tomoki-fujita@otaru-ekisaikai.jp

Dr. Tsutomu Nishida

Department of Gastroenterology, Toyonaka Municipal Hospital

4-14-1 Shibaharacho, Toyonaka-shi, Osaka, Japan

Telephone: +81-6-6843-0101 Email: tnishida.gastro@gmail.com

Dr. Katsuhiro Mabe

Department of Gastroenterology, National Hospital Organization Hakodate Hospital 18-16 Kawaharachou, Hakodate-shi, Hokkaido, Japan

Telephone: +81-138-51-6281 Email: kmabe@hnh.hosp.go.jp

Dr. Tetsuya Sumiyoshi

The Center for Digestive Disease, Tonan Hospital

Kita 1, Nishi 6, Chuo-ku, Sapporo-shi, Hokkaido, Japan

Telephone: +81-11-231-2121 Email: t-sumiyoshi@tonan.gr.jp

Dr. Kenkei Hasatani

Department of Gastroenterology, Fukui prefectural hospital

2-8-1, Yotsui, Fukui-shi, Fukui, Japan

Telephone: +81-776-54-5151 Email: hasatani9@yahoo.co.jp

Dr. Tatsuya Mikami

Division of Endoscopy, Hirosaki University Hospital

53, Honcho, Hirosaki-shi, Aomori, Japan

Telephone: +81-172-39-5053 Email: tmika@hirosaki-u.ac.jp

Dr. Kazuo Hara

Department of Gastroenterology, Aichi Cancer Center Hospital

1-1 Kanokoden, Chikusa-ku, Nagoya-shi, Aichi, Japan

Telephone: +81-52-762-6111 Email: khara@aichi-cc.jp

Dr. Tetsuro Honda

Department of Gastroenterology, Nagasaki Harbor Medical Center City Hospital

6-39, Shinchimachi, Nagasaki-shi, Nagasaki, Japan

Telephone: +81-95-822-3251 Email: tetsupeacevo@yahoo.co.jp

Dr. Shu Kiyotoki

Department of Gastroenterology, Shuto General Hospital

1000-1 Kogaisaku, Yanai-shi, Yamaguchi, Japan

Telephone: +81-820-22-3456 Email: shu2026eb@hi.enjoy.ne.jp

Dr. Katsumi Yamamoto

Department of Gastroenterology,  Japan Community Healthcare Organization Osaka Hospital

4-2-78, Fukushima, Fukushima-ku, Osaka, Japan

Telephone: +81-6-6441-5451 Email: katsumiy770@yahoo.co.jp

**Biostatistician**

Tomohiro Shinozaki

Department of Biostatistics, School of Public Health, the University of Tokyo

7-3-1 Hongo, Bunkyo-ku, Tokyo, Japan

Telephone: +81-3-3815-5411 Email: shinozaki@epistat.m.u-tokyo.ac.jp

**Data Coordinating Center and Manager**

Dr. Munenori Takata

Mariko Takeda

Department of Clinical Research Support Center, The University of Tokyo Hospital

7-3-1 Hongo, Bunkyo-ku, Tokyo, Japan

Telephone: +81-3-5800-9762 Email: takatam-tky@umin.ac.jp

**Medical Monitor**

Yumi Tanaka

Department of Clinical Research Support Center, The University of Tokyo Hospital

7-3-1 Hongo, Bunkyo-ku, Tokyo, Japan

Telephone: +81-3-5800-9762 Email: tanakaym-tky@umin.ac.jp

**Regulatory Specialist Regrading Safety**

Ikue Wada

Clinical Research Support Center, The University of Tokyo Hospital

7-3-1 Hongo, Bunkyo-ku, Tokyo, Japan

Telephone: +81-3-5800-9762 Email: iwada-tky@umin.ac.jp

**Audit**

Yuki Kusaka

Department of Clinical Research Governance, The University of Tokyo Hospital

7-3-1 Hongo, Bunkyo-ku, Tokyo, Japan

Telephone: +81-3-5800-9051 Email: ykusaka-tky@umin.ac.jp

**Independent Effect Judgment Committee**

Dr. Shiro Oka

Department of Endoscopy, Hiroshima University Hospital, Hiroshima, Japan

1-2-3 Kasumi, Minami, Hiroshima-shi, Hiroshima, Japan

Telephone: +81-82-257-5555 Email: oka4683@hiroshima-u.ac.jp

Dr. Hirotsugu Watabe

Wakamiya Watabe Clinic

3-3-16, Wakamiya, Ichihara-shi, Chiba, Japan

Telephone: +81- 436-43-0609 Email: hwatabegi@gmail.com
